# Research on the Mechanical Response and Constitutive Model of 18Ni300 Manufactured by SLM with Different Build Directions

**DOI:** 10.3390/ma17174246

**Published:** 2024-08-28

**Authors:** Zhenchao Liang, Qing Zhang, Wenbin Li, Weihang Li

**Affiliations:** Ministerial Key Laboratory of ZNDY, Nanjing University of Science and Technology, Nanjing 210094, China; 122101222049@njust.edu.cn (Z.L.);

**Keywords:** mechanical properties, selective laser melting, constitutive model, SHPB, 18Ni300

## Abstract

Metals manufactured by selective laser melting (SLM) with different directions exhibit different mechanical properties. This study conducted dynamic and static mechanical tests using a universal testing machine and split-Hopkinson bar (SHPB). The mechanical properties of 18Ni300 with 0° and 90° build directions manufactured by SLM were compared, and the micro-structure properties of the two build directions were analysed by metallographic tests. The Johnson–Cook (J-C) constitutive model was fitted according to the experimental results, and the obtained constitutive parameters were verified by numerical simulations. The results revealed that the constitutive model could predict the mechanical properties of 18Ni300 in a dynamic state. The build direction had little influence on the mechanical properties in a static state, but there was a significant difference in the dynamic state. The difference in the dynamic compressive yield strength of the 18Ni300 material manufactured by SLM with two build directions was 9.8%. The SLM process can be improved to produce 18Ni300 with uniform mechanical properties by studying the reasons for this difference.

## 1. Introduction

With the development of three-dimensional printing technology, many fields have witnessed the emergence of new applications [1,2,3,4,5]. Among these, selective laser melting (SLM) technology has already opened up new applications in several areas. For example, the aerospace division of Honeywell in the United States has utilized this technology to manufacture heat exchangers and metal brackets [1]. Airbus also employs SLM to produce engine pylon hinge brackets for its A320 series aircraft. In the medical field, SLM technology is currently used to manufacture dental crowns and bridges from Co-Cr alloys [2]. The use of titanium (Ti) in the medical field is well established, and medical equipment and supplies made from titanium have now started to incorporate SLM technology [3].

Studies have assessed the microstructure and mechanical characterizations of various materials in the field of SLM [6,7,8,9,10]. Krakhmalev et al. [6] studied the mechanical characterizations, porosity, and microstructure of 16Ti6Al4V prepared by SLM under controlled oxygen conditions. The twin phenomenon in the tensile test was found by electron diffraction and a transmission electron microscopy analysis. According to this analysis, the possible cause of the twinning is thermal stress regulation in the manufacturing process. The microscopic defect analysis shows that the coalescence of the pores is the main crack formation mechanism leading to the final fracture of the prestrain sample, which has the typical characteristics of a cup and cone fracture. Liverani et al. [7] studied the construction of AISI316L manufactured with different process parameters. The microstructure, test defect properties, and mechanical properties were studied. The results show that near-full-density specimens with better elongation at break values and a higher ultimate tensile strength can be obtained. Zheng Wang et al. [8] systematically studied the optimization of process parameters of Ti6Al4V alloy during SLM and investigated the relationship between mechanical properties and microstructure. The study found that the influences of laser power and scanning velocity on surface roughness and porosity are contradictory. Appropriately reducing the scanning speed and increasing the laser power help to decrease the surface roughness while improving the dimensional accuracy and density of the material. When the laser power is set at 200 W, as the scanning speed increases from 250 mm/s to 850 mm/s and then to 1750 mm/s, the microstructure undergoes a transformation from equiaxed grains to a mixture of equiaxed and columnar grains, and finally to fully columnar grains.

Unlike parts forged into shape, parts manufactured using three-dimensional printing may not have uniform mechanical properties in all directions. The mechanical properties of different build directions may also vary [11,12,13]. Maconachie et al. [11] studied the mechanical characterization of AlSi10Mg with different orientations, especially under dynamic and static conditions. They found that samples perpendicular to the construction direction showed greater levels of ductility than samples in other directions. Three specimens with different build directions were manufactured using SLM, and quasi-static and split-Hopkinson bar tests were used to analyse the tensile properties at strain rates ranging from 3.33 × 10^−2^ to 2.4 × 10^3^ s^−1^. The results show that there is little difference in strength between the samples with different construction directions, but the samples perpendicular to the construction direction have better levels of ductility. Wang et al. [12] studied the tension and compression asymmetry and anisotropy of 316L prepared by SLM in different directions by combining experiments with a crystal plastic finite element (CPFE) simulation. The results show that the non-uniform grain distribution leads to the plastic anisotropy of the material during stretching and compression; that is, there are significant differences in stress levels under different loading modes. By comparing the experimental data with the model prediction results, it is found that CPFE simulation can qualitatively predict the real stress–strain behaviour under uniaxial tensile and uniaxial compression. Brandl et al. [13] researched the crack behaviour, fatigue properties, and microstructure of AlSi10Mg alloy samples prepared by adding additives. The samples were prepared by SLM and subsequently machined. In the study, 91 samples were manufactured, tested at room temperature, and heated (300 °C) from different directions. The specimens were compared in a peak hardening (T6) state and an untreated state. The results showed that the subsequent heat treatment had a remarkable influence on the anti-fatigue performance of the sample, but the effect on the building direction of the specimen is relatively small.

The application of metallic materials in engineering structures relies on the research and establishment of constitutive models. The Johnson–Cook model, a constitutive model of materials, can characterize the mechanical performance changes of materials in states with an high strain rate, at a high pressure and a high temperature. Many researchers have assessed the constitutive models of materials based on SLM printing in this area [14,15,16,17,18,19,20,21]. Li et al. [14] studied the influence of various scanning speeds on the mechanical properties of 316L. They used four different scanning speeds (350 mm/s, 650 mm/s, 950 mm/s, and 1250 mm/s) to prepare the sample by selective laser melting (SLM) technology. The research team evaluated the dynamic quasi-static and compressive mechanical characterizations of these four specimens through dynamic and static mechanical tests. In addition, they analysed the differences in mechanical properties between the samples through microscopic observations. The results show that the samples prepared by these four processes show mechanical properties of obvious strain rate strengthening and typical viscoplastic characteristics. In the meantime, the scanning speed has a significant effect on the internal defect and molten pool characteristics of the sample, and the yield strength of the sample also declines remarkably with the reduction in molten pool characteristics. In the end, the modified J-C model more accurately describes the mechanical characterizations of 316L manufactured by SLM. Zheng et al. [15] studied the impact of scanning strategies in the anisotropic characterizations of the Ti-6Al-4V alloy. They employed different scanning strategies at angles of 0°, 67.5°, and 90° to fabricate samples and analysed the anisotropic effects of these scanning strategies on various aspects, including surface morphology, microstructure, and microhardness, as well as dynamic and quasi-static mechanical properties. Based on the consequence of the dynamic and quasi-static compression experiments, the researchers also developed an improved J-C constitutive model. Aktürk et al. [16] established a study on the constitutive parameters of the AlSi10Mg alloy, aiming to determine the J-C model through high-temperature tensile tests. In the quasi-static tensile tests, three different strain rates were selected: 10^−3^ s^−1^, 10^−2^ s^−1^, and 5 × 10^−2^ s^−1^, and the experiments were performed at room temperature. For the high-temperature tensile tests, the study considered testing at three temperatures: 24 °C, 150 °C, and 300 °C, with a reference strain rate of 10^−3^ s^−1^. Finally, the research team compared the numerical simulation results of the J-C model with the experimental data obtained, finding that the maximum error between the two was approximately 7.5%.

In recent years, 18Ni300 has been studied more and more in SLM [22,23,24,25,26,27,28]. Chaolin Tan et al. [22] studied the structure and properties of 18Ni300 prepared by SLM and aged at 490 °C for 6 h. The results show the surface roughness of the parts is 4–5 μm. The lower surface roughness of the part indicates that the moulding process has a higher manufacturing accuracy. Due to precipitation strengthening, the hardness and ultimate tensile strength of the heat treatment are increased, but the elongation at break is decreased. These are caused by the increased brittleness of the material. Mariusz Król et al. [23] studied the influences of different process data on the porosity, hardness, and microstructure of SLM 18Ni300. It was found that the reversal of martensite to austenite and the precipitation of metal compounds occurred during the heating cycle. The martensite phase transition occurs during the cooling process. After an aging treatment at 460 °C for 5 h, the hardness of the material was also improved. Jun Song et al. [24] studied the mechanical properties and microstructure of 18Ni300 prepared by SLM during the remelting process. Under a certain combination of parameters, large pores are successfully eliminated, and the density is improved. A further study found that a remelting treatment had no effect on the tensile strength but increased the ductility and microhardness.

In this study, the dynamic and static mechanical responses of 18Ni300 in two construction directions of 0° and 90° were studied by quasi-static compression, high-temperature compression, and Hopkinson bar tests, and the microscopic morphology of the material under the two construction directions was compared by observing the metallographic structure. According to the mechanical results, the J-C model of the material under two construction directions was fitted, and the Hopkinson bar test was verified by LS-DYNA. This work may serve as an engineering reference for the application and simulation of SLM printing 18Ni300 in high-strain-rate and high-temperature environments.

## 2. Materials and Methods

### 2.1. Specimen Manufacturing Method

**Figure 1:**
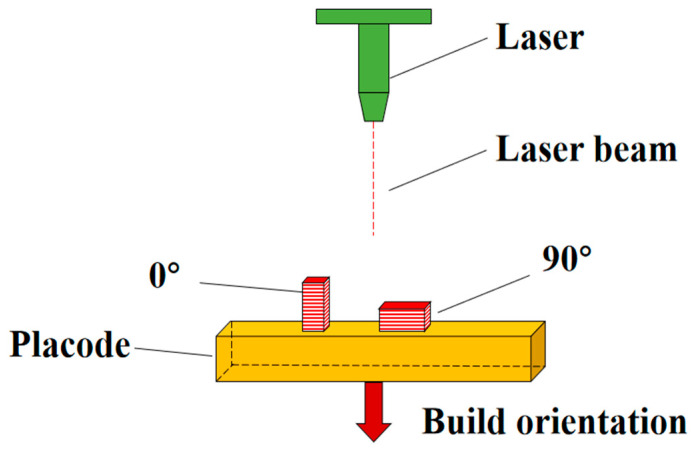
Specimen manufactured by SLM.

**Figure 2:**
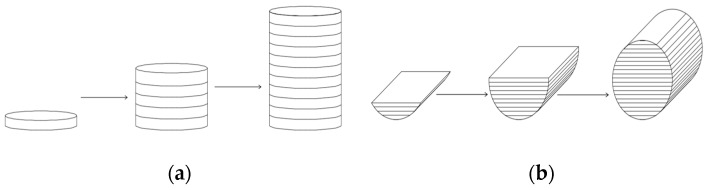
Fabrication process of the specimens with two build directions: (**a**) generation process of the 0° build direction specimen; (**b**) generation process of the 90° build direction specimen.

SLM is a complicated process, with parts affected by thermal effects including heat concentration, repeated melting, and rapid solidification. The manufacturing results of this process are influenced by processing parameters, material characteristics, tool paths, and component shapes. This structure will fundamentally differ from that of parts manufactured using traditional methods. The specimens constructed in this study were manufactured by SLM and were all cylindrical, as shown in Figure 1. The build direction was divided into the 0° build direction and 90° build direction. In the context of additive manufacturing, the 0° build direction referred to the direction in which the printed layers were aligned parallel to the axis of the cylindrical end surface of the specimen, resulting in a 0° angle between the layers. Similarly, the 90° build direction referred to the orientation in which the printed layers were aligned perpendicular to the axis of the cylindrical end face, resulting in a 90° angle between the layers, as shown in Figure 1. It is difficult to intuitively understand the process of printing the test pieces; thus, the process of printing the 0° and 90° test pieces is illustrated, as shown in Figure 2.

The nearly spherical 18Ni300 powder provided by ACME Company in China was prepared using the gas atomization method. The chemical composition of the 18Ni300 powder used for sample fabrication is shown in Table 1.

A total of 30 specimens with dimensions φ5 × 6 were produced using two different build orientations. Additionally, 20 specimens with dimensions φ4 × 2 were created using the same two build orientations. All 18Ni300 alloy specimens sized φ5 × 6 and φ4 × 2 were manufactured using an SLM280 machine made by ACME from Changsha, China. This system is equipped with a wavelength of 1064 nanometres and a fibre-modulated pulsed laser with a maximum power of 500 watts. The SLM parameters for preparing the 18Ni300 alloy in this study were provided by ACME, and the parameters of process are shown in Table 2.

### 2.2. Equivalent Static Compression

The importance of specimen size in the quasi-static compression experiment was self-evident. The size of the specimens directly affected the reliability of the test and the accuracy of the results, since the size of the specimen affected the stress distribution, deformation characteristics, and final failure mode. In quasi-static compression test of 18Ni300, the specimen size was devised according to the Chinese national standard [25] “Compression test Method for Metal Materials at Indoor Temperatures”, in which the shape of the specimens was cylindrical. On the basis of the Chinese national standard, the ratio of length *l* to diameter *d* of the specimen was 1 < *l*/*d* < 2, and the design size of the quasi-static compression test specimen was ø5 × 6 mm. The test machine consisted of an MTS370.50 instrument (MTS from Eden Prairie, MN, USA) for which the loading rates of the test were 0.36, 3.6, and 36 mm/min, and the corresponding strain rates were 1 × 10^−3^ s^−1^, 1 × 10^−2^ s^−1^, and 1 × 10^−1^ s^−1^. The maximum load range of MTS370.50 is ±100 kN, and the load measurement accuracy is ±0.5%. The dynamic frequency range is 0~80 Hz.

The corresponding deformation and the testing machine load were obtained from the data collected during the experiment. The engineering stress–strain data for the specimen is calculated using the following equation [22]:

(1) $$\left\{\begin{array}{l} \sigma =\frac{4P}{\pi d_{0}^{2}}\\ \varepsilon =\frac{l_{0}-l}{l_{0}}=\frac{\Delta l}{l_{0}}=1-\frac{l}{l_{0}} \end{array}\right.$$

where *P* is the testing machine load, *d*_0_ is the initial diameter of the test piece, *l* and *l*_0_ denote the instantaneous length and initial length of the test piece, respectively, Δ*l* is the time deformation, and *σ* and *ε* denote the engineering stress and engineering strain of the test piece, respectively.

To obtain the true stress–strain curve of the specimen, the engineering stress–strain curve was obtained according to the following equation:

(2) $$\left\{\begin{array}{l} \sigma_{T}=\frac{P}{A}=\frac{P}{A_{0}}\times \frac{l}{l_{0}}=\frac{P}{A_{0}}\left(1-\varepsilon \right)=\sigma \left(1-\varepsilon \right)\\ \varepsilon_{T}=\int_{l}^{l_{0}}\frac{dl}{l}=\ln\left(\frac{l_{0}}{l}\right)=-\ln(1-\varepsilon ) \end{array}\right.$$

where *σ_T_* and *ε_T_* are the true stress and strain, respectively. This relation was also applicable to the processing of high-temperature compression test and SHPB test data, in which *A*_0_ and *A* denote the initial and instantaneous cross-sectional areas of the specimen, respectively.

### 2.3. High-Temperature Compression Test

The testing machine consisted of an MTS370.50 instrument. Before the test, the high-temperature furnace was preheated to the required temperatures of 348, 448, and 548 K. The high-temperature furnace was then opened, and the test pieces were placed between the two test stands. The test stand was adjusted to the appropriate position, and the high-temperature furnace was heated, for which the strain rate was 1 × 10^−3^ s^−1^ and the loading velocity was 0.36 mm/min. The true stress–strain curve can be obtained similarly to quasi-static compression through Equations (1) and (2).

### 2.4. SHPB Test

The SHPB test is commonly used in the field of dynamic mechanics due to its simple test principle and facile data acquisition. In this study, the length of the bullet was 0.4 m, the lengths of the incident rod and the projection rod were 1.5 m, and the diameter of the rod was 14.5 mm. The material consisted of 18Ni300.

**Figure 3:**
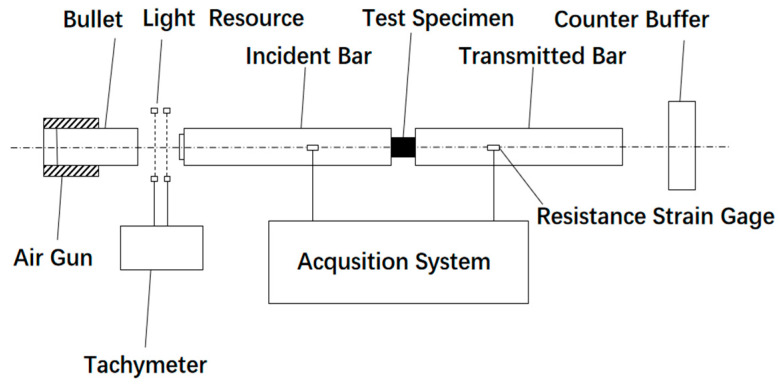
SHPB device diagram.

SHPB utilizes the following principle. A bullet fired by an air gun hits the incident rod after passing through the velocity meter, and one-dimensional compressive stress wave propagating to the specimen is generated in the incident rod. After passing through the specimen and the projection rod, a reflected wave and a transmitted wave will be generated in the incident rod. By installing resistance strain gauges on two rods, the voltage signals of incident waves, reflected waves, and transmitted waves can be obtained. After data processing, these signals will be transformed into experimental results. The SHPB device is shown in Figure 3.

For the SHPB test, the diameter of the sample is generally 80% of the rod diameter, so that when the transverse expansion diameter of the sample reaches the rod diameter, the real axial strain can reach 30%. After analysing the errors caused by longitudinal and radial inertia, Davies and Hunter found that the size of the appropriate sample should meet the following condition:

(3) $$\frac{l_{0}}{d_{0}}=\sqrt{\frac{3\upsilon_{s}}{4}}$$

where *l*_0_ and *d*_0_ denote the diameter and length of the specimen, and *υ_s_* is the Poisson’s ratio. The Poisson’s ratio of steel is about 0.3. At this point, the length-to-diameter ratio of the sample was the closest to 0.5, and the error caused by the inertia effect was the smallest. The final sample size was Ø4 × 2 mm. The SHPB test was designed to obtain the stress–strain curves of materials with different strain rates.

The true stress–strain curve obtained by processing the data obtained from the test is given as follows [29]:

(4) $$\left\{\begin{array}{l} \sigma =\frac{AE}{A_{0}}\varepsilon_{t}\\ \varepsilon =-\frac{2c_{0}}{l_{0}}\int_{0}^{t}\varepsilon_{r}d\tau \\ \dot{\varepsilon }=-\frac{2c_{0}}{l_{0}}\varepsilon_{r} \end{array}\right.$$

where *c*_0_ is the elastic wave speed in the rod, *ε*, *ε_r_*, and *ε_t_* denote the rod strain corresponding to the independent propagation of the incident wave, reflected wave, and transmitted wave, and *l*_0_ is the length of the specimen. A and E denote the area and elastic modulus of cross profile of the bar, respectively.

### 2.5. Metallographic Test

The samples were polished using MC004-350W (WHW from Shanghai, China) and German-Leica DM2000x (Leica from Wetzlar, Germany) polishing machines. Then, the samples were polished using PG-1 metallographic sample polishing machine (CSOIF from Shanghai, China). Last, the samples were observed with Zeiss-Axio Imager2 (ZEISS from Oberkochen, Germany), Zeiss lab.a1 China -9XB-PC optical microscopes (ZEISS from Oberkochen, Germany) after corrosion via aqua regia. Hydrochloric acid content was 22.8~25.8%, and nitric acid content was 2.5–3.1%.

## 3. Results and Discussion

### 3.1. The Result of Quasi-Static Compression Test

**Figure 4:**
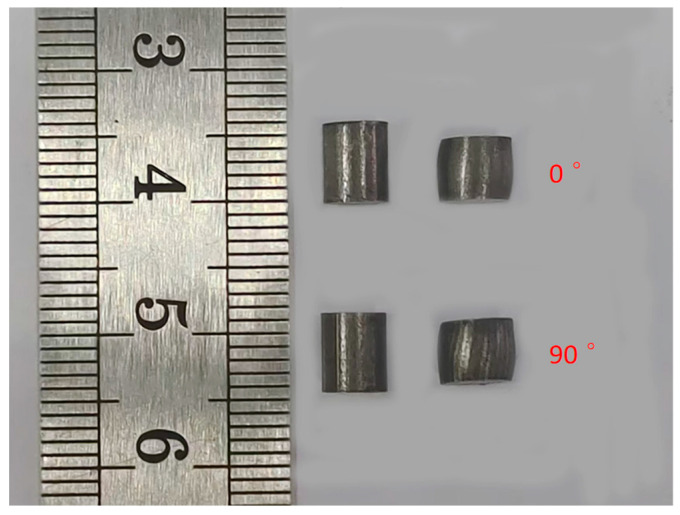
The deformation degree of the sample.

**Figure 5:**
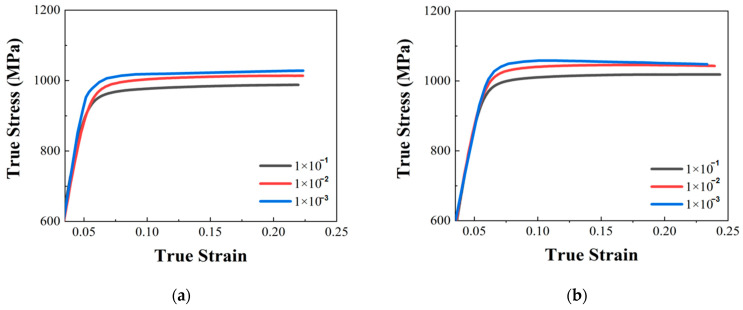
True stress–strain curve under quasi-static compression test with two build directions: (**a**) true stress–strain curve of 18Ni300 fabricated in the 0° direction; (**b**) true stress–strain curve of 18Ni300 in 90° direction.

The specimens with the two build directions were grouped, and repeated effective tests were used at three strain rates. The data obtained from the three tests were averaged to obtain the true stress–strain curve of the 18Ni under the corresponding deformation conditions. The deformation degree of the sample is shown in Figure 4. The true stress–strain curves are as shown in Figure 5.

**Figure 6:**
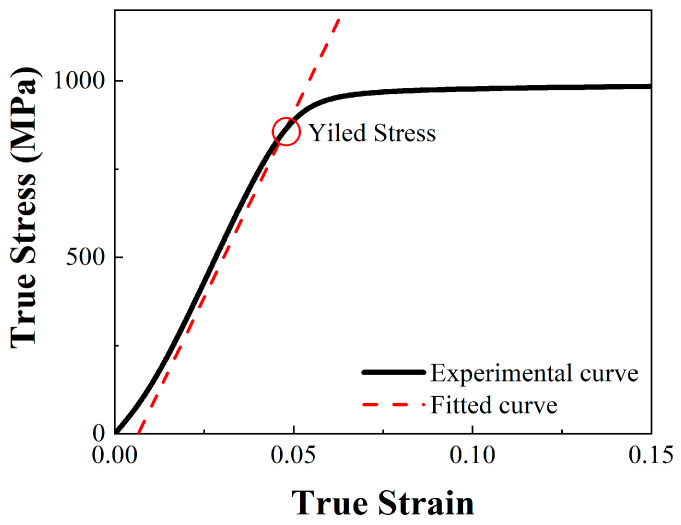
Determination of yield strength.

As shown in Figure 5, 18Ni300 with the two build directions showed no evident yield point in the quasi-static compression test. Therefore, as shown in Figure 6 and Table 3, the stress value at *σ*_0.2_ could be taken as the compressive yield strength. This method could also be used to obtain the yield strength using the SHPB and high-temperature quasi-static compression test. The yield point of quasi-static compression test are as shown in.

### 3.2. The Result of High-Temperature Compression Test

**Figure 7:**
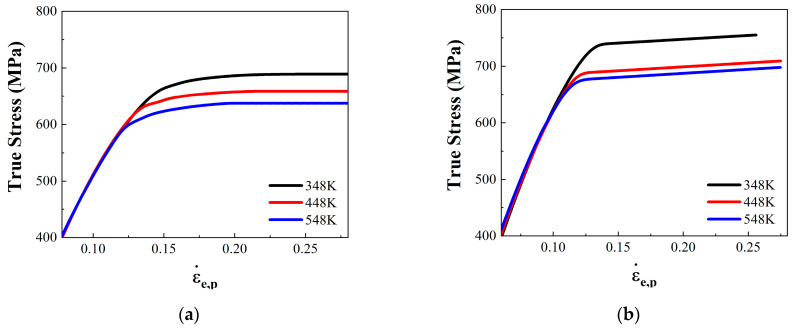
True stress–strain curves of the high-temperature compression tests: (**a**) True stress–strain curve of 18Ni300 in the 0° direction; (**b**) true stress–strain curve of 18Ni300 in the 90° direction.

The true stress–strain curves of the high-temperature compression tests are as shown in Figure 7. According to this calculation method of yield stress introduced in Section 4.1, the compressive yield strength of 18Ni300 with the two build directions at three temperatures was obtained, and the results are shown in Table 4.

### 3.3. The Result of SHPB Test

**Figure 8:**
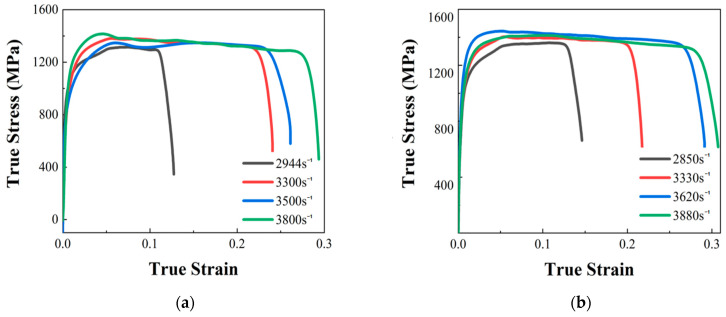
True stress–strain curves of SHPB of 18Ni300 at different strain rates: (**a**) SHPB test results of 18Ni300 in the 0° build direction with different strain rates; (**b**) SHPB test results of 18Ni300 in the 90° build direction with different strain rates.

The SHPB test results of 18Ni300 for the two build directions are shown in Figure 8. The determination method of the yield point is as shown in Figure 6, which was used to analyse the curves in Figure 8. The yield points of the SHPB test with the two build directions of 18Ni300 were obtained, and the results are as shown in Table 5.

### 3.4. The Result of Metallographic Test

**Figure 9:**
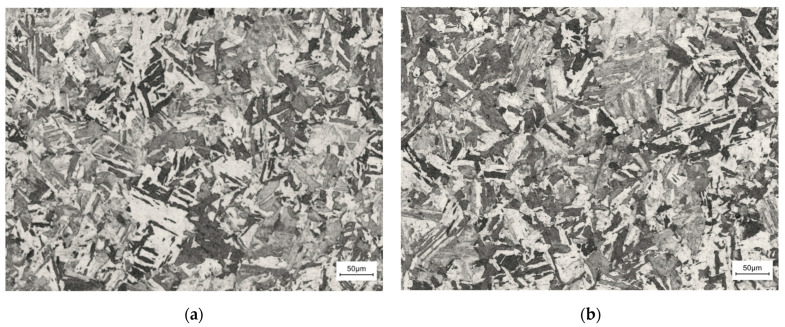
Micro-structure of the 18Ni300 specimen with two build directions: (**a**) micro-structure of the 18Ni300 specimen for the 0° build direction; (**b**) micro-structure of the 18Ni300 specimen for the 90° build direction.

As shown in Figure 9, the presence of slab martensitic is dominant due to the fast cooling rate. In addition, residual austenite and pearlite can be observed. The grain shape is mainly columnar. By comparing the metallography micro-structures with the two build directions, we observed that slat martensitic phases were present in the specimens with both build directions. Compared to the micro-structure of the specimens with two build directions, we found that the specimen for the 90° build direction had more martensitic phases than the specimen for the 0° build direction.

## 4. Discussion

### 4.1. Strain Hardening Effect

**Figure 10:**
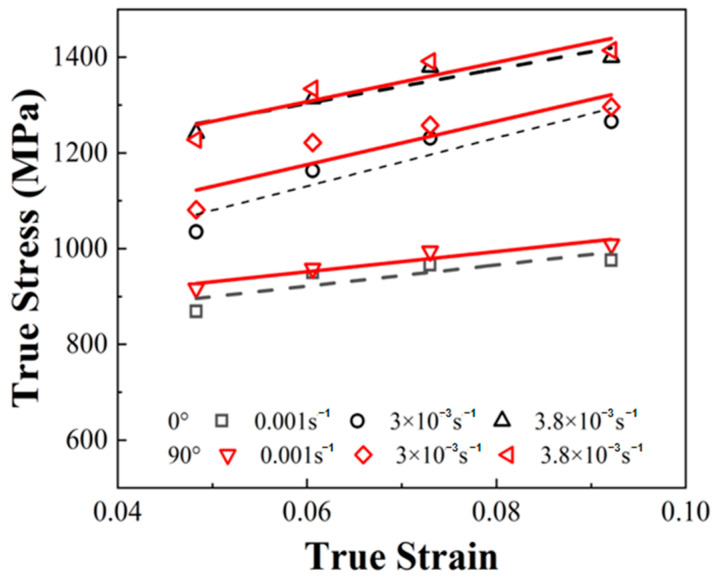
Strain hardening effect of flow stress at indoor temperature (298 K).

The flow stress changes with the strain of the materials for the two build directions are shown in Figure 10 and Table 6. The flow stress at 90° was significantly higher than that at 0°. The two directions exhibited strain hardening behaviour at an indoor temperature (298 K). With an increase in the deformation amount, the deformation stress inside the material obviously increased, resulting in an increase in flow stress. Compared to the quasi-static deformation, the strain hardening effect with the dynamic deformation was more obvious. The strain hardening behaviour in the 90° direction was more obvious than that in the 0° direction.

With a strain rate of 0.001 s^−1^ and a deformation condition of 298 K, the equivalent plastic strain increased from 0.047 to 0.093, and the flow stress of the specimen utilizing the 0° build direction increased from 869 to 976 MPa, resulting in an increase of 12.3%. The flow stress of the specimen utilizing the 90° build direction increased from 916 to 1009 MPa, resulting in an increase of 9.2%. When the deformation strain rate increased to 3000 s^−1^, the flow stress of the specimen utilizing the 0° build direction increased from 1035 to 1265 MPa, resulting in an increase of 22.2%. The flow stress of the specimen utilizing the 90° build direction increased from 1081 to 1296 MPa, resulting in an increase of 19.8%. When the deformation strain rate increased to 3800 s^−1^, the flow stress of the specimen utilizing the 0° build direction increased from 1242 to 1400 MPa, resulting in an increase of 12.7%. The flow stress of the specimen utilizing the 90° build direction increased from 1227 to 1414 MPa, resulting in an increase of 15.2%. 

### 4.2. Strain Rate Strengthening Effect

**Figure 11:**
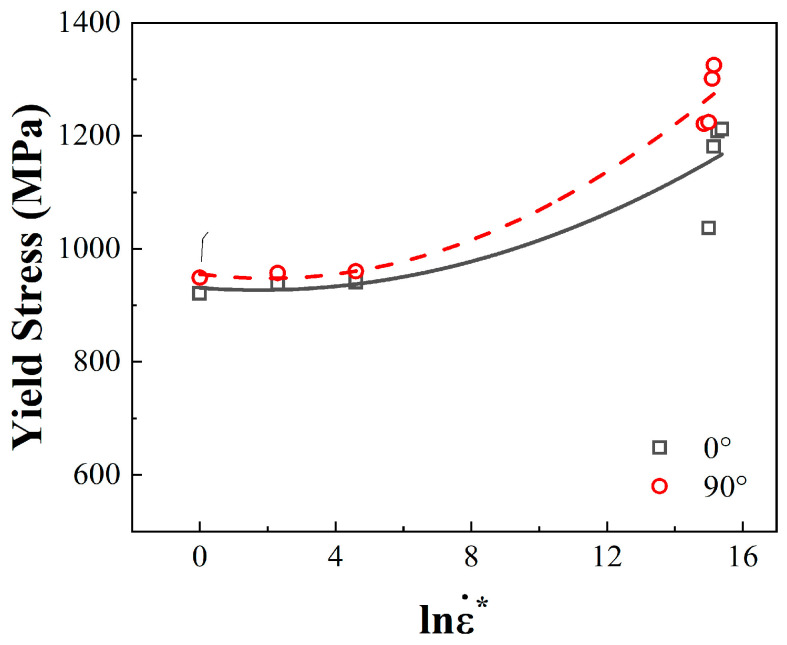
Variation in yield strength with increasing strain rate.

On the basis of the thermal activation theory of the plastic deformation of metal materials, with the increase in the applied strain rate, the energy required and the internal stress experienced by the material in the deformation process will increase, resulting in a rise in thermal stress. The variations in yield strength with the strain rate in two directions at the indoor temperature (298 K) are as shown in Figure 11. The 18Ni300 materials utilizing both build directions showed obvious strain rate strengthening behaviour. At a larger strain rate range, the yield strength of the 18Ni300 exponentially rose with an increase in the logarithm of the strain rate. The rising trend of the yield strength was low in the quasi-static state. In the dynamic deformation state, 18Ni300 utilizing the 90° build direction demonstrated more obvious strain rate strengthening behaviour.

### 4.3. Thermal Softening Effect

**Figure 12:**
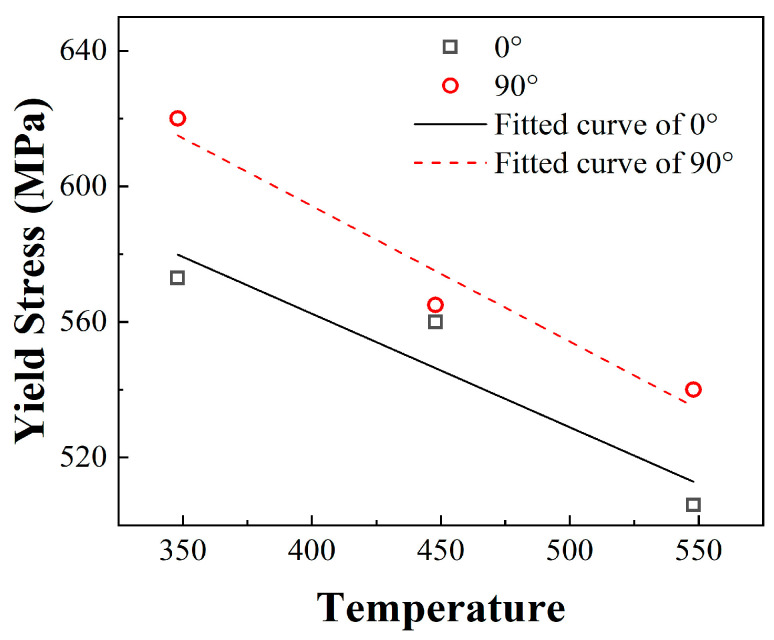
Yield strength variations with temperature.

The phenomenon of increasing deformation temperature leading to a reduction in the flow stress of a material is known as the thermal softening behaviour of materials. Heat softening is an important property of homogeneous metallic materials. Notably, the plastic deformation of metals will be affected by the increase in deformation temperature with high strain rates. The variation law of yield strength of the two build directions at an ambient temperature was analysed, and the results are as shown in Figure 12.

As shown in Figure 12, the yield strength of the materials in the two build directions decreased with an increase in ambient temperature. In addition, both materials exhibited a thermal softening effect. The yield strength of 18Ni300 for the 90° build direction at high temperatures was significantly higher than that of 18Ni300 for the 0° build direction.

### 4.4. Microstructure Characterization

Martensite is critical for strengthening steel parts, as it can improve their strength and hardness [30]. And the grains of the specimen with the 90° build direction are coarser than those of the 0° build direction. This results in a higher strength for the specimens for the 90° direction. This was the reason for the difference between 18Ni300 for the two build directions, in terms of dynamic mechanical properties.

## 5. J-C Constitutive Model and Verification

### 5.1. J-C Constitutive Model

The application of 18Ni300 in engineering structure is inseparable from the research and establishment of its constitutive model. For the constitutive model of ultra-high-strength materials, a model must be able to characterize the mechanical properties of metals at high temperatures, high pressures, and high strain rates. The J-C model comprehensively considers the effects of strain strengthening, strain rate, and temperature on the mechanical properties. Due to its simple expression form and easy access to model parameters, this model has been widely used in engineering research. The concrete form of the constitutive model is given as follows [30]:

(5) $$\sigma =(A+B\varepsilon_{p}^{n})(1+C\ln{\dot{\varepsilon }}^{*})(1-{T^{*}}^{m_{jc}})$$

where *A*, *B*, *C*, *n*, and *m_jc_* serve as material constitutive parameters with certainty, ${\dot{\varepsilon }}^{*}=\frac{\dot{\varepsilon }}{{\dot{\varepsilon }}_{0}}$, $T^{*}=\frac{\left(T-T_{r}\right)}{\left(T-T_{m}\right)}$, *T_r_* is the reference temperature, and *T_m_* is the melting point of the material.

Compared to other models, the J-C constitutive model offers the advantages of having a simple function form, easy calibration of its constitutive parameters, and easy engineering application. This model takes into account the effects of strain hardening, the strain rate effect, and the temperature effect. The J-C model describes the interaction among the three by introducing three independent terms and couples their interaction effects by multiplying these three terms together. Among these, the J-C model can be characterized by the flow stress *σ_pl_* of the material with respect to the hardening behaviour of the plastic strain through Equation (6). The softening behaviour of the yield strength *σ_y_* with the temperature was characterized by Equation (7), and the strengthening behaviour of the yield strength *σ_y_* with the strain rate was characterized by Equation (8). The strengthening of the yield strength of 18Ni300 relative to the strain rate was linear according to this J-C model, which is not the case according to Figure 11:

(6) $$\sigma_{pl}=A+B\varepsilon_{e,p}^{n}$$

(7) $$\sigma_{y}=A(1-{T^{*}}^{m_{jc}})$$

(8) $$\sigma_{y}=A(1+C\ln{\dot{\varepsilon }}^{*})$$

Compared to the linear simplified form of the J-C model, Huh et al. [31] proposed a single quadratic strain rate correlation term that could describe the exponential variation law of the material yield strength with the logarithm of the strain rate. Therefore, by modifying the strain rate effect term of the J-C constitutive model, the function form of the modified J-C model could be obtained as follows:

(9) $$\sigma =(A+B\varepsilon_{p}^{n})(1+C_{1}\ln{\dot{\varepsilon }}^{*}+C_{2}\ln{\dot{\varepsilon }}^{*2})(1-{T^{*}}^{m_{jc}})$$

where *A*, *B*, *C*_1_, *C*_2_, *n*, and *m_jc_* denote the undetermined constitutive parameters, while the other parameters remained the same as those in the J-C model. The constitutive parameters of 18Ni300 were obtained in the function form of Equation (9), utilizing the following steps.

### 5.2. Result of Fitting Parameter

The static compression test can be used to obtain *A*, *B*, and *n*. At the reference temperature (298 K) and reference strain rate (1 × 10^−3^ s^−1^), we obtain the following:

(10) $$\sigma =A+B\varepsilon_{e,p}^{n}$$

**Figure 13:**
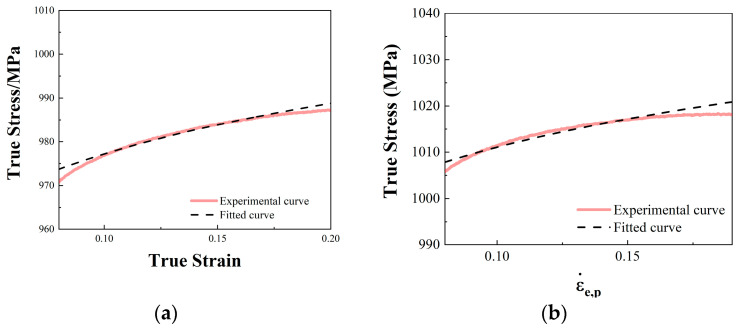
A, B, and n fitting results of 18Ni300 under two build directions: (**a**) *A*, *B*, and *n* fitting results of 18Ni300 for the 0° build direction; (**b**) *A*, *B*, and *n* fitting results of 18Ni300 for the 90° build direction.

The yield strength in Table 3 was taken as the value of *A*, where the stress–strain curve of the plastic deformation section of the quasi-static test was converted into the ln(*σ-A*) − ln*ε_e_*_,*p*_ curve. The intercept was ln*B* and the slope was *n* of the resulting curve. The fitting curve was obtained from Equation (10), and the fitting curve was matched with the experimental data points, as shown in Figure 13.

According to the SHPB test results provided in Section 3.2.

**Figure 14:**
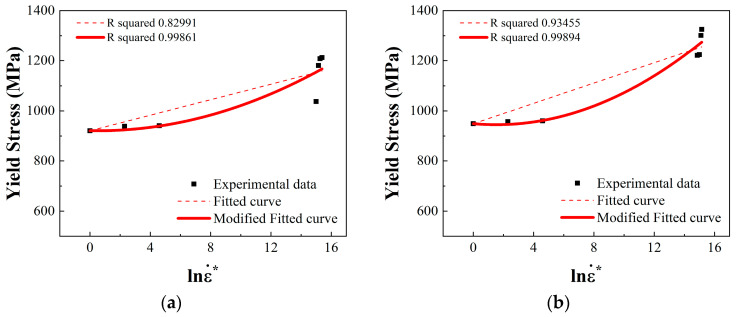
Fitting of constitutive parameter C of 18Ni300 for two build directions: (**a**) fitting result of constitutive parameter C of 18Ni300 for the 0° build direction; (**b**) fitting result of constitutive parameter C of 18Ni300 for the 90° build direction.

The fitting curve was obtained from Equation (9), and a comparison between the fitting curve and test data is shown in Figure 14. From the figure, it can be seen that the fitting degree of this one-dimensional quadratic strain-rate-related term is higher than that of the original strain-rate-related term, indicating that this form of fitting is more accurate.

At the reference strain rate (1 × 10^−3^ s^−1^), we obtain the following:

(11) $$\sigma =A+B\varepsilon_{e,p}^{n}$$

**Figure 15:**
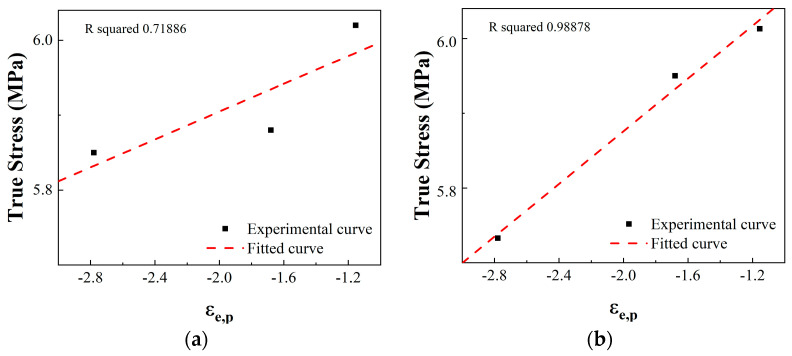
Fitting curve of *m_jc_* for two build directions: (**a**) fitting curve of *m_jc_* for the 0° build direction; (**b**) fitting curve of *m_jc_* for the 90° build direction.

The fitting curve of *m_j_*_c_ is shown in Figure 15.

### 5.3. Finite Element Modelling

#### 5.3.1. Numerical Modelling

**Figure 16:**
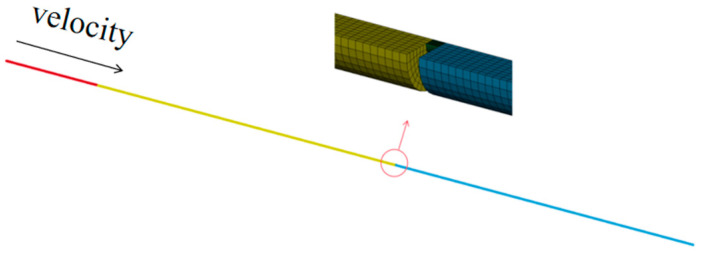
Finite element simulation model of SHPB.

The SHPB rod material model are as shown in Table 7. The initial value of the J-C model was obtained by fitting the existing data, as shown in Table 8. To verify the degree of preciseness of the J-C model, this paper adopted the commercial finite element software LSDYNA (Version 11.0) to simulate and verify the SHPB test [32]. The following simulation model was created. To improve the calculation efficiency, a one-quarter-scale finite element model of SHPB consistent with the test was established, as shown in Figure 16. The element type was SOLID164, and the symmetry plane was set as symmetric constraints. The contact type between the rods and between the rod and the specimen was automatic surface-to-surface contact.

The plastic hardening material model was used for the SHPB rod. Table 8 lists the model parameters, and the material model of the specimen was *MAT_JOHNSON_COOK.

#### 5.3.2. Analysis of the Numerical Simulation Results

**Figure 17:**
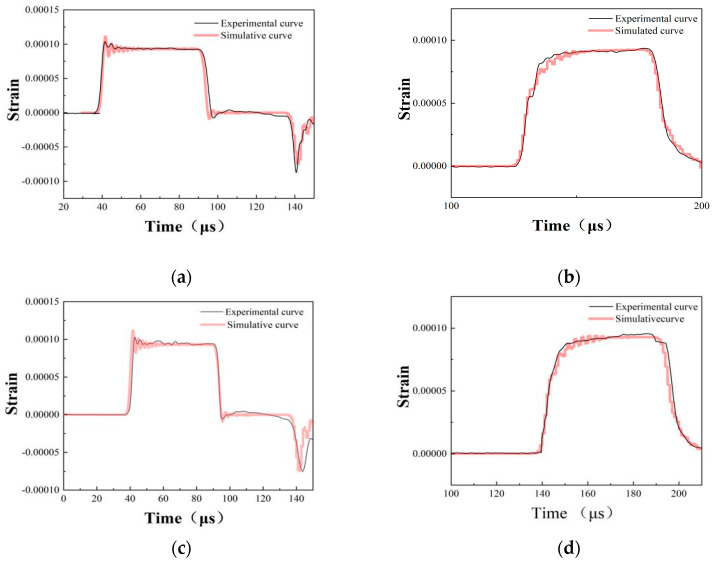
Comparison of the numerical simulation results with the experimental results: (**a**) strain–time curve of SHPB entrance rod for the 0° build direction; (**b**) strain–time curve of the SHPB transmission rod for the 0° build direction; (**c**) strain–time curve of the SHPB entrance rod for the 90° build direction; (**d**) strain–time curve of the SHPB transmission rod for the 90° build direction.

In the SHPB verification test, the impact rod struck the incident rod at a certain speed to load the specimen. The relationship between the impact velocity and strain rate is as shown in Table 9. From Figure 17, it can be seen that the experimental data are consistent with the numerical simulation data. The maximum error between the experimental data and the simulation data is about 8.9%, indicating that the J-C model can predict the mechanical properties of 18Ni300 manufactured by SLM under dynamic conditions.

## 6. Conclusions

By conducting several experiments and numerical simulations, this study assessed the difference in the mechanical properties of 18Ni300 with two build directions, and the micro-structures of 18Ni300 with two build directions were compared. The results were obtained through quasi-static compression, SHPB, and high-temperature compression tests, using the J-C model of 18Ni prepared by SLM, which has two build orientations. Numerical simulation modelling utilizing LSDYNA was carried out, and the SHPB test was simulated according to the J-C constitutive model parameters. The experimental data were compared with the numerical simulation results. The numerical simulations predicted the mechanical properties of 18Ni300 manufactured by SLM with dynamic state accuracy. The following main conclusions were drawn from this study.

(1) By comparing the stress–strain curves at two construction directions in a static state, we found that 18Ni300 with the two build directions did not show yield points, and the 18Ni300 material with both build directions demonstrated a thermal softening effect. Moreover, 18Ni300 exhibited a strain rate strengthening effect for both build directions. However, the yield strength of 18Ni300 does not show significant differences in the two build directions. 18Ni300 exhibited strain hardening and strain rate strengthening effects for both build directions. However, the yield strength of 18Ni300 for the two build directions demonstrated certain differences under the four strain rates. Specifically, the yield strength of 18Ni300 for the 90° build direction was 9.6% higher than that of 18Ni300 for the 0° build direction. By comparing the micro-structures of 18Ni300 manufactured by SLM for the two build directions, we found that 18Ni300 for the 90° build direction contained more and bigger martensite phases than 18Ni300 for the 0° build direction, according to the microstructure. This was the reason for the difference in mechanical properties of the 18Ni300 material with the two build directions.
(2) A J-C constitutive model of 18Ni300 with different construction directions was obtained, and the SHPB test was simulated according to the J-C parameters. The strain rate–time curve obtained from the SHPB simulation matches well with the experimental results. Comparing the numerical simulation results and the experimental results, the maximum error between the experimental and simulated results was around 8.9%. This proves the J-C model can precisely predict the mechanical properties of 18Ni300 under dynamic conditions.

In this study, the mechanical properties of 18Ni300 under two construction directions are compared and analysed. In the future, the SLM process can be improved to produce 18Ni300 with uniform mechanical properties by studying the reasons for this difference. The obtained J-C constitutive model can provide reference for the numerical simulation of 18Ni300 under dynamic conditions in the future.

## Figures and Tables

**Table 1:** Chemical composition of the 18Ni300 powder.

Ni	Ti	Co	Al	Mo	Si	Cr	Mn	C	Fe
17.70	0.72	9.05	0.077	4.79	0.025	0.031	0.022	0.007	67.578

**Table 2:** Product process.

Material	Layer Thickness (mm)	Laser Power (W)	Scanning Speed (mm/s)	Heat Treatment Method
18Ni300	0.05	200	1000	Maintained at 900 °C for 2 h and cooled below 80 °C

**Table 3:** Compressive yield strength of 18Ni300 for two build directions.

Build Direction	Strain Rate (s^−1^)	Compression Yield Stress (MPa)
0°	1 × 10^−3^	921
	1 × 10^−2^	939
	1 × 10^−1^	941
90°	1 × 10^−3^	949
	1 × 10^−2^	957
	1 × 10^−1^	960

**Table 4:** Yield points of 18Ni300 for two build directions at three temperatures.

Build Direction	Temperature (K)	Compression Yield Stress (MPa)
0°	348	573
	448	560
	548	506
90°	348	620
	448	565
	548	540

**Table 5:** Dynamic yield point of 18Ni300 for two build directions.

Build Direction	Strain Rate (s^−1^)	Compression Yield Stress (MPa)
0°	2944	1037
	3300	1181
	3500	1208
	3800	1212
90°	2850	1221
	3330	1224
	3620	1301
	3880	1325

**Table 6:** Analysis of the strain hardening effect.

Build Direction	Strain Rate (s^−1^)	Changes in Flow Stress (MPa)	Rate of Increase
0°	1 × 10^−3^	869–976	12.3%
	3 × 10^−3^	1035–1265	22.2%
	3.8 × 10^−3^	1242–1400	12.7%
90°	1 × 10^−3^	916–1009	9.2%
	3 × 10^−3^	1081–1296	19.8%
	3.8 × 10^−3^	1227–1414	15.2%

**Table 7:** Split-Hopkinson pressure bar rod material model.

*ρ* (g/cm^3^)	Elasticity Modulus *E* (GPa)	Yield Stress *σ_S_* (MPa)	Poisson’s Ratio *v*
8.2	210	1900	0.33

**Table 8:** Modified J-C constitutive model parameters.

Build Direction	*A*	*B*	*n*	*C*_1_	*C*_2_	*m_jc_*
0°	921	112	0.303	0.00117	0.00120	0.0928
90°	949	100	0.209	0.00134	0.00259	0.1762

**Table 9:** Relationship between impact velocity and strain rate.

Build Direction	Striking Velocity (m/s)	Strain Rate (s^−1^)
0°	6.36	2944
	8.22	3300
	8.44	3500
	9.26	3800
90°	7.01	2850
	8.26	3330
	9.31	3620
	10.01	3880

## Data Availability

The data presented in this study are unavailable due to privacy about the material manufacturer.
